# Renal vein and inferior vena cava thrombosis as a rare complication of pyelonephritis

**DOI:** 10.1016/j.jvscit.2025.101875

**Published:** 2025-06-09

**Authors:** Jan Wirth, Karel Houdek, Jiří Moláček, Lukáš Bednář

**Affiliations:** a Department of Surgery, Faculty of Medicine in Pilsen, Charles University, Pilsen, Czech Republic; b Department of Vascular Surgery, University Hospital in Pilsen, Pilsen, Czech Republic

**Keywords:** Renal vein thrombosis, Acute pyelonephritis, Nephrectomy, Cavotomy, Thromboinflammation

## Abstract

Renal vein thrombosis (RVT) and inferior vena cava thrombosis are extremely rare conditions, yet potentially life-threatening complications of pyelonephritis. This report describes the case of a 66-year-old patient with undiagnosed pyelonephritis, who subsequently developed RVT with an extension to the inferior vena cava. The management of the condition involved a multidisciplinary approach that resulted in nephrectomy with an extraction of the thrombus through cavotomy. This case report emphasizes the necessity of early recognition of RVT owing to pyelonephritis and complex management of this condition to prevent the adverse outcomes of impending complications.

Renal vein thrombosis (RVT) in adults is associated commonly with hypercoagulable states originating in nephrotic syndrome or malignant diseases.^1^^,^^2^ RVT is managed commonly by anticoagulant therapy, and sometimes by endovascular or open surgical procedures.^3^ An infectious etiology of RVT, such as pyelonephritis, is an extremely rare complication, with only a few cases documented in the literature.^2^, ^3^, ^4^, ^5^ Here we present a unique case of untreated pyelonephritis complicated by RVT resulting in nephrectomy, highlighting the importance of considering a potential RVT in patients with pyelonephritis who present atypical symptoms. Written consent was obtained from the patient.

## Case report

A 66-year-old woman with a history of pulmonary embolism on anticoagulant therapy (apixaban), and immune thrombocytopenia came to our hospital with complaints of recent hematuria, nausea, and back and right flank pain persisting for three days with a normal body temperature of 36.8 °C. Initial clinical and sonographic evaluations revealed no significant pathology. No laboratory tests were conducted except for urine cultures, and no antibiotics were administered. The patient was discharged with a reduced dose of apixaban from 5 mg twice per day to just once per day, and with etamsylate tablets. The presence of hematuria was attributed to the anticoagulant therapy.

However, the patient returned 5 days after being discharged with blood clots in urine and with worsening back and flank pain. Clinical examination revealed tenderness in the right flank (Giordano's sign) and pain on palpation in the lower right quadrant of the abdomen. A bedside ultrasound scan showed a hypotonic right ureter and abnormal echogenicity of the renal parenchyma, with no evidence of urinary tract obstruction or RVT. Urinary catheterization was performed, and massive hematuria with thrombus in the urine drainage bag was observed. Laboratory tests showed a white blood cell count of 24.40 × 10^9^/L, elevated C-reactive protein at 227 mg/L, a platelet count of 89 × 10^9^/L, a creatinine level of 192 μmol/L, a sodium level of 125 mmol/L, and a chloride level of 93 mmol/L. A noncontrast computed tomography scan revealed signs of pyelonephritis on the right side.

**Fig 1:**
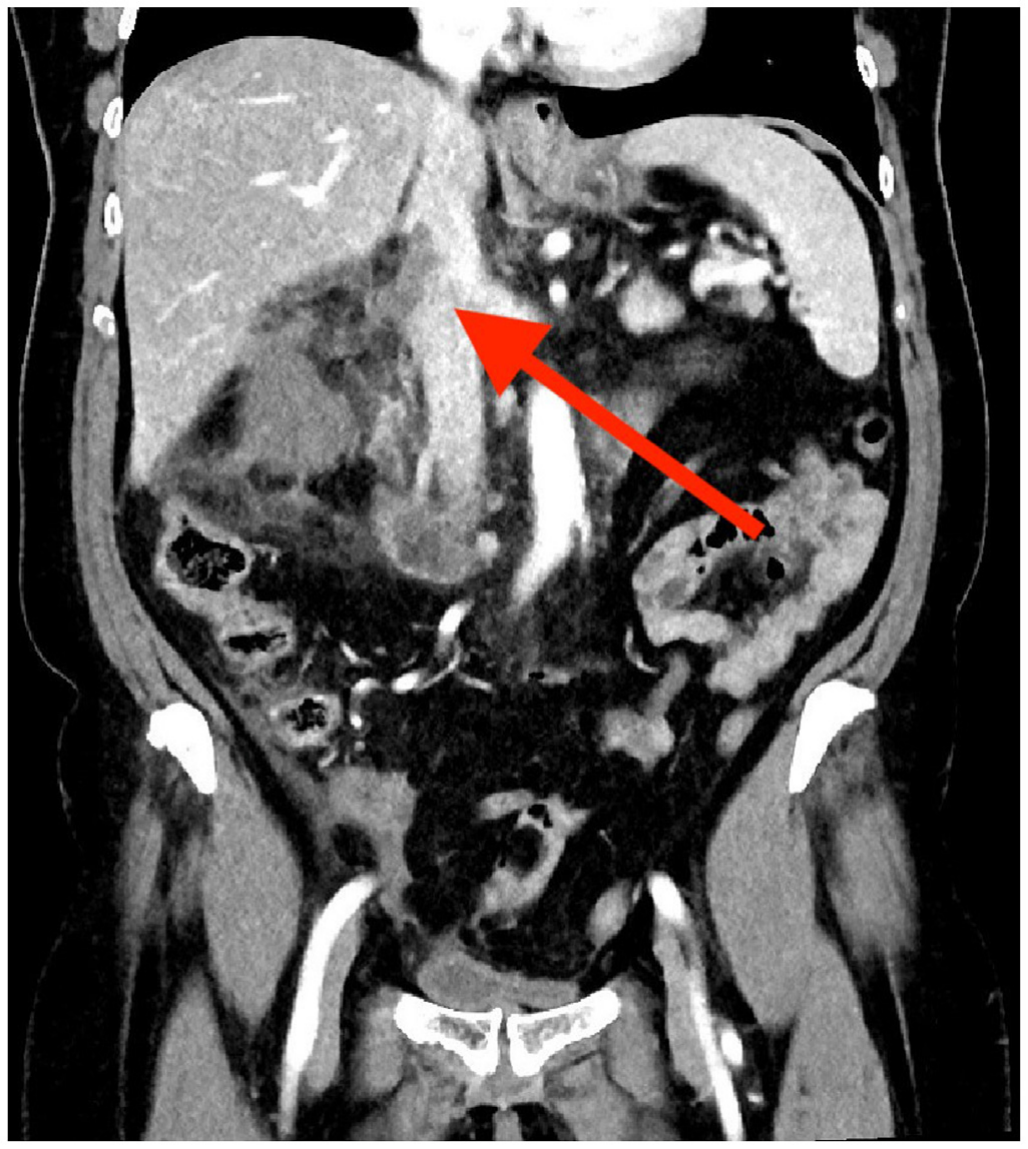
Thrombus extending to the inferior vena cava (IVC) (*red arrow*).

**Fig 2:**
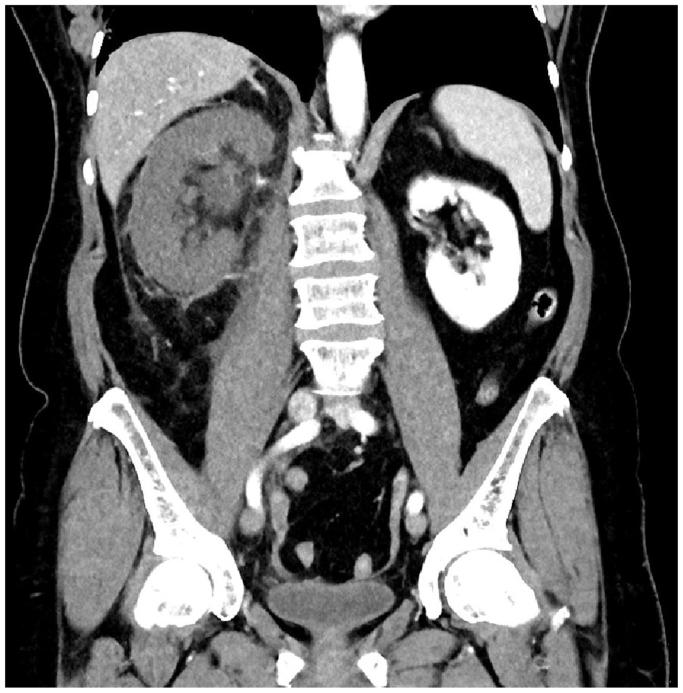
Right kidney infarction secondary to renal vein thrombosis (RVT).

The patient was admitted to the urology department. Multiple blood and urine cultures were obtained, but no pathogen was identified. The patient was given antimicrobial treatment with cefotaxime 2 g intravenously every 8 hours with the reduced dosage of apixaban; etamsylate was discontinued. On day 3 after admission, a computed tomography scan showed infarction of the right kidney, a thrombus in the right renal vein protruding into the inferior vena cava (IVC), and no excretion of contrast agent (Figs 1 and 2). A multidisciplinary team decided on surgical treatment, and the patient was transferred to the surgical department.

In the operating room, a right subcostal incision was performed, and the IVC with the right kidney was accessed through a right medial visceral rotation. An approximately 10-cm segment of the IVC with the left renal vein was visualized. The ureter and the renal artery were ligated, and a tangential occlusion clamp was utilized on the IVC, the renal vein ostium was excised, and the kidney was removed. A short cavotomy was performed, the thrombus was removed gently, and the defect was oversewn with running suture. Renal function was soon normalized, and there were no complications after the procedure. Histological examination of the kidney showed multiple thrombi in the veins and destruction of the renal pelvis by pyelonephritis. Thrombophilia screening was performed, and all tests were negative.

## Discussion

RVT secondary to pyelonephritis is a rare condition that requires early recognition and intervention. The most common causes of RVT are currently nephrotic syndrome or malignancy—both of which lead to a hypercoagulable state.^3^ Severe infections such as pyelonephritis can lead to venous thrombosis through a phenomenon called thromboinflammation, which is a complex process based on an interaction between proinflammatory factors and the immune system's response.^3^ The most common causative agents are *Escherichia coli* and *Klebsiella pneumoniae*.^4^

RVT can be present acutely with symptoms resembling pyelonephritis or remain undetected until a complication draws attention to it, often with devastating consequences. It should be especially suspected in patients treated for pyelonephritis who are not responding to the therapy. The symptoms may mimic those of a urinary tract obstruction or pyelonephritis, which can include flank or diffuse abdominal pain, flank tenderness upon percussion, hematuria, and rapid deterioration of renal function in patients with nephrotic syndrome. Furthermore, RVT of the left renal vein can lead to gonadal vein thrombosis, being present with symptoms of pelvic congestion syndrome in females or as a painful swelling of the left testis in males.^1^^,^^6^ Ultrasound signs of RVT include renal enlargement with loss of corticomedullary differentiation and absent flow in the renal vein. Doppler assessment of renal veins should be attempted, although it could be challenging technically.^7^

There are no standardized guidelines for the treatment of RVT. Management typically depends on the clinical presentation, renal function, and the extent of the thrombosis. In such cases of complicated pyelonephritis, the prescription of anticoagulant medication is usually for a duration of 6 to 12 months or until renal function has been restored; antibiotics are recommended for a period of 4 to 6 weeks.^3^^,^^8^ In cases of renal function deterioration, a more invasive approach to the therapy should be considered in addition to anticoagulation. Endovascular interventions have been shown to improve renal function and thrombus clearance more effectively when combined with anticoagulant therapy compared with anticoagulant therapy alone.^8^ However, endovascular interventions carry up to a 20% risk of pulmonary embolism. To mitigate this risk, the use of a temporary IVC filter has been recommended in cases of RVT extending to the IVC, with early retrieval after the procedure.^9^^,^^10^ In cases of RVT, nephrectomy is performed typically when there is evidence of kidney necrosis with a risk of rupture. This risk was present in our patient; therefore, the decision was made to perform an open surgical procedure. The surgical intervention and exposure depend on the extent of thrombosis and can vary from simple ligation of the renal vein at the ostium with nephrectomy up to liver mobilization and cardiopulmonary bypass.^11^ Open surgical thrombectomy of the renal vein has been replaced by catheter-directed lysis, although it may still be used in the case of RVT in a recently operated kidney owing to the risk of hemorrhage with lytic agents.

In this case, we hypothesize that the failure to diagnose and treat the initial episode of pyelonephritis led to the development of intraparenchymal venous thrombosis. A decrease in her apixaban and the addition of etamsylate may have increased the likelihood of a prothrombotic state. When she returned 5 days later, there was no initial evidence of RVT on imaging; however, intrarenal thrombi could not be excluded. The lack of aggressive anticoagulation at this time owing to the presence of gross hematuria led to extension of the thrombus into the main renal vein and IVC and renal infarction, despite initiation of antibiotic therapy.

## Conclusions

Patients who present with pyelonephritis and hematuria and demonstrate unresponsiveness to standard therapy should be considered at high risk for RVT. The risks of full anticoagulation should be balanced against the loss of renal function to avert severe subsequent complications, such as life-threatening pulmonary embolism or renal loss.

## Funding

This work was supported by the Cooperation Program, within the research area of Surgical Disciplines.

## Disclosures

None.
